# Live surgical broadcasts: a scoping review

**DOI:** 10.1093/bjsopen/zraf051

**Published:** 2025-05-27

**Authors:** Essam Rama, Vikas Khanduja

**Affiliations:** School of Clinical Medicine, University of Cambridge, Cambridge, UK; School of Clinical Medicine, University of Cambridge, Cambridge, UK; Young Adult Hip Service, Department of Trauma and Orthopaedics, Addenbrooke's Hospital, Cambridge University Hospitals NHS Foundation Trust, Cambridge, UK

Live surgery has been a valuable educational tool since the anatomical demonstrations of more than 500 years ago^1^. Improvements in audiovisual and teleconferencing technology have facilitated the emergence of live surgical broadcasts (LSBs), which involve high-quality streaming of operating room procedures to both global audiences and the in-house conference hall viewership at live surgery events. Proponents highlight the interactive nature of LSBs, which allow participants to engage with expert surgeons as they demonstrate new techniques, manage complications, and navigate challenging steps in real time^2^. However, the practice has not been without controversy, raising ethical and safety concerns that challenge its continued implementation^3^.

A scoping review was conducted to provide a comprehensive overview of the research in this field. Inclusion and exclusion criteria are presented in *Table S1*. Studies written in English or with an available English translation were included; studies before 2005 were excluded. In all, 36 studies were included in the scoping review. The PRISMA diagram for study inclusion is shown in *Fig. 1*. Included studies were classified into four categories: the impact on patients, the impact on surgeons, the impact on the audience, and alternatives to live surgery. There was significant overlap between categories, as shown in *Table S3*. These impacts are summarised in *Table S4*.

**Fig. 1:**
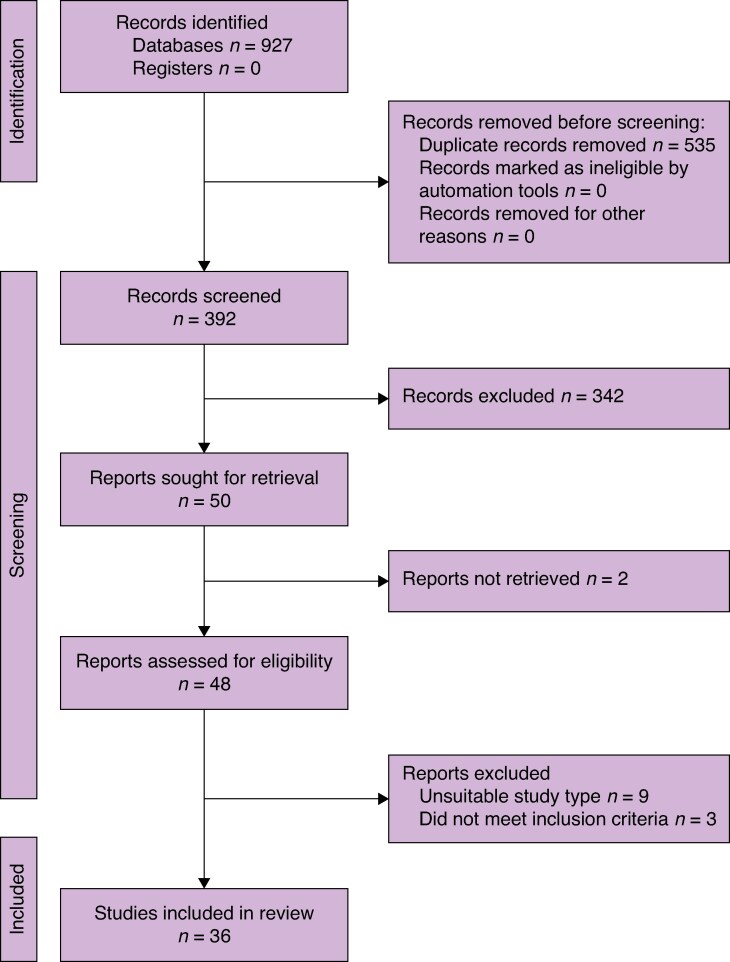
PRISMA flow diagram for included studies A detailed search strategy is provided in *Table S2*. The EMBASE, MEDLINE, and PubMed databases were searched for eligible articles.

LSBs provide an unfiltered educational experience, allowing observers to witness and interact with expert surgeons making decisions in real-time. Surveys indicated that live broadcasts are highly valued by attendees for their ability to enhance clinical and anatomical understanding. For example, 91% of urologists who attended LSBs reported the experience as a ‘great way’ to educate^4^. The reported benefits were learning new techniques^5^, avoiding complications, and improving surgical skills. LSBs fostered interaction between surgeons and audiences, increased engagement and provided knowledge^2^ that would otherwise be difficult to share.

The potential risks to patients remain key points of contention. Studies have generally found that complication rates for live surgeries are comparable to those of routine procedures, although some studies concerning bariatric and colorectal surgery have reported a higher complication rate, a longer operative time, and increased blood loss (*Table S5*).

Surveys of audience members and surgeons emphasised the additional pressures placed on surgeons, which may compromise performance (*Table S6*). Performing in unfamiliar environments, working with unfamiliar teams, and the need to narrate and interact with a live audience introduced additional challenges that could affect outcomes. Ethical concerns also arise regarding informed consent, confidentiality, and potential financial conflicts of interest, particularly when LSBs are sponsored by surgical device companies.

It has been suggested that a comparable educational benefit may be derived from alternatives to live surgery that focus on patient safety first and reduce the associated risks for the patient and the additional challenges faced by the surgeons and surgical team^6^. An ‘as-live’ surgical broadcast (ALSB) refers to the use of prerecorded unedited videos, where the interaction between surgeons is maintained without the element of distraction and with the ability to replay key parts of the operation, maximizing learning for the audience^6^.

Studies comparing LSBs and ALSBs have yielded mixed results^6,7^. Surveys suggested ALSBs provide similar educational value. ALSBs address several ethical concerns by enabling procedures to be performed in the surgeon’s home institution, minimizing unfamiliarity and ensuring continuity of care for patients.

The dilemma of live surgery is whether the educational benefit outweighs the potential for patient harm. The future of live surgical education will likely involve a blend of live and prerecorded approaches, balancing the educational benefits of LSBs with the safety and ethical advantages of alternatives. Advances in simulation technology and virtual reality may further enhance surgical training, providing immersive, risk-free environments that complement live and prerecorded broadcasts.

## Supplementary Material

zraf051_Supplementary_Data

## Data Availability

Data will be made available upon request.
