# Supplementary material for: Live surgical broadcasts: a scoping review
Source: BJS Open. 2025 May 27;9(3):zraf051. doi: 10.1093/bjsopen/zraf051 (PMC12107351; doi:10.1093/bjsopen/zraf051)
Supplement: zraf051_Supplementary_Data [file zraf051_supplementary_data.docx]

**Title: Live Surgical Broadcasts: scoping review**

**Authors**

Essam Rama^1^, Vikas Khanduja^2^

^1^University of Cambridge School of Clinical Medicine, Cambridge, United Kingdom

^2^Addenbrooke's Hospital, Cambridge University Hospitals NHS Foundation Trust, Cambridge, United Kingdom.

**Corresponding author.**

**Name and address:** Essam Rama, University of Cambridge School of Clinical Medicine, Cambridge, United Kingdom

**ORCID ID:** 0000-0003-3928-0575

**Twitter:** @EssamRama_

**Supplementary Materials - Index**

| **Supplementary Methods** | *Page 2* |
| --- | --- |
| **Supplementary Results** |  |
| Impact on patients | *Page 3* |
| Impact on surgeons | *Page 5* |
| Impact on the audience | *Page 5* |
| The future of live surgery | *Page 7* |
| **Supplementary Figures and Tables** |  |
| Table S1 | *Page 9* |
| Table S2 | *Page 10* |
| Table S3 | *Page 11* |
| Table S4  Table S5 | *Page 13*  *Page 14* |
| Table S6 | *Page 17* |
| **References** | *Page 20* |
|  |  |

**Supplementary Methods**

This review was conducted based on the PRISMA extension for scoping reviews (PRISMA-ScR) guidelines(1) and Arksey and O’Malley’s five-stage methodological framework for scoping reviews(2). Inclusion and exclusion criteria were determined using the PICOS model (population, intervention, comparison, outcome, study type)(3), as shown in **Supplementary Table 1 (Table S1).** Studies written in English or where a translation was available were included, studies before 2005 were excluded**.** To identify relevant papers, the following bibliographic databases were searched from inception to September 2023: MEDLINE, Ovid EMBASE, and PubMed. The search included variations of the following terms: live, live-streaming, surgery, outcomes, and complications; the full search strategy is shown in **Supplementary Table 2 (Table S2)**. One reviewer screened studies based on the inclusion and exclusion criteria. Initial screening of the title and abstract was followed by screening of the full text. Reasons for exclusion were identified at each stage.

Data from eligible studies were charted using a standardised abstraction tool designed for this study. The data charting form was updated iteratively throughout the charting process. Data from this study were extracted into a google sheets spreadsheet. We abstracted data on article characteristics (title, author, year, study design) , study population, and the main outcomes of each study (e.g. patient outcomes, outcomes of surveys stratified by domain).

Following data extraction, included studies were classified into four categories: impact on patients, the impact on surgeons, the impact on the audience, and alternatives to live surgery. There was significant overlap between categories, as shown in **Supplementary Table 3 (Table S3)**. These impacts are summarised in **Supplementary Table 4 (Table S4)**. When we identified a systematic review, we determined if studies that potentially met our inclusion criteria had been missed by our search.

**Supplementary Results**

A total of 927 articles were identified after the initial search from the three databases. No additional articles which met our inclusion criteria were identified from the three available systematic reviews(4–6). 392 articles remained for title and abstract screening after deduplication. 342 articles were excluded, leaving 50 articles for full-text screening, of which 36 were eligible for this review.

## Impact on patients

Eighteen studies focused on patient outcomes during or after live surgical procedures. Outcomes included but were not limited to: perioperative complications classified according to the Clavien-Dindo grade (e.g. constipation, pulmonary embolism, urinary tract infection, pleural effusion, tension pneumothorax, bleeding, stroke, and mortality), operative time, length of hospital stay, and any requirement for revision surgery. Patient outcomes are shown in **Supplementary Table 5 (Table S5).**

Eight of these eighteen studies made comparisons with the available literature, with six studies (five urology(8,9,16,31,35), one cardiac(37)) finding complication rates with live surgery to be comparable (no difference) to the available literature. In contrast, Lafosse et al. demonstrated lower complication rates in live shoulder surgery compared to the available literature(18), whereas Ruiz de Gordejuela et al. noted the opposite: with higher morbidity and reoperation rates in individuals undergoing live bariatric surgery(33).

The remaining ten studies made direct comparisons with control groups. Of these, nine studies (six urology(19,22,23,25,26,29), colorectal(32), liver(30), ophthalmology(28)) reported no difference in complication rates between groups of patients undergoing live surgery and those undergoing routine procedures. Ogaya-Pinies et al. reported a shorter robotic console time for the live surgery group(25), whereas Legemate et al. observed longer operative times for their ureterorenoscopic live surgery group compared to controls(19). Similarly, Ratti et al. observed a longer operating time for live laparoscopic liver resections compared to conventional elective procedures(30). Puzo et al. only found a greater loss of endothelial cell density in live cataract surgery compared to non-live surgery when using toric intraocular lenses(28). Furthermore, despite observing no difference in perioperative complications, Ramírez-Backhaus et al. found higher rates of positive margins at final pathology in patients who underwent live radical prostatectomy, compared to controls(29). In contrast, Unal et al. observed higher rates of overall complications, longer operative times, greater intraoperative estimated blood loss, and longer hospital stays for patients undergoing minimally invasive live colorectal surgery, compared to their control group(38).

Ten surveys included questions on the impact on patients and/or the ethics of live surgery for patients. Of these, five surveys targeted participants at conference meetings(12,14,20,27,36), two surveys were aimed specifically at surgeons who perform LSB(17,34), and three surveys were sent en masse to professional associations of: ophthalmologists(15), paediatric surgeons(10), and urologists(11).

There is no clear consensus of *opinion* on whether live surgery is more harmful than conventional elective procedures. For example, Dingemann et al. reported surgeons and attendees ‘agreed that live surgery *may* be harmful’(10), yet Duty et al. found only 11.5% of surgeons to be worried for their patients’ well-being during live surgery(11). Likewise, 68% of surveyed UK ophthalmologists perceived the complication rate of live surgery to be higher than the background rate(15), but 67.1% of surveyed urologists felt they ‘never’ had more complications while performing live surgery outside their home institution(17).

The willingness of respondents to allow family members to undergo live surgery further informs of the perceived risk associated with live surgery. Duty et al. reported that only 28.2% of urologists would let a visiting faculty member operate on them or a family member(11), Dingemann et al. found 36% of surgeons would not consent for live surgery for their own child(10). Notably, Phan et al. found that the number of unwilling respondents for LSB decreased from 60% preconference to 32% after the conference, highlighting the potential for preconceptions to be altered with positive experiences(27). Similarly, Elsamra et al. reported 58% of respondents would allow themselves to be a patient of a live operation(12).

The perception of the risk associated with live surgery is not independent from the perception of whether live surgery is ethical. For instance, 79% of moderators who regarded live surgery as ethical would allow themselves or a family member to be a patient, compared to 25% of those who did not regard it as ethical(34). Furthermore, Duty et al. and Elsamra et al. found 71% and 78% of respondents deemed live surgery to be ethical, respectively(11,12). In the latter, those who thought live surgery was unethical perceived live surgery to have a lower benefit compared to those who thought it was ethical. In contrast, 83% of ophthalmologists believed live surgery to not be in the patient's best interests, in accordance with the aforementioned 68% who perceived a higher complication rate(15).

Three studies compared live surgery with semi-live or as live surgery. Finch et al. found a lower concern that patient outcomes may have been compromised with ALSB compared to LSB and respondents were less likely to recommend a family member to be a patient or be a patient themselves in a LSB setting compared to ALSB(14). Legemate et al. found respondents to perceive a higher complication risk for live surgery than semi-live surgery and a greater proportion of respondents felt patient safety was often not the highest priority during surgery in live cases (15%) compared to semi-live cases (8%)(20). However, only a third of urologists felt semi-live broadcasts are ethically less concerning than LSBs(36).

## Impact on surgeons

Eight surveys assessed the impact of performing live surgery on surgeons(10–12,14,15,17,20,34). Important domains included: the impact of pressure and anxiety on performance, the appropriateness of the surgical procedure chosen, challenges inside the operating theatre, and challenges from outside the operating theatre as a result of the live broadcast. The findings are summarised in **Supplementary Table 6 (Table S6)**.

## Impact on the audience

Sixteen studies assessed the impact of live surgical broadcast on the audience. A large proportion of these considered the benefits for surgical education and training. For example, Dingemann et al. reported that live surgery was rated as educationally essential by both surgeons and attendees and that surgeons, but not attendees, perceived the educational benefits to outweigh the disadvantages for patients(10). Likewise, 91% of urologists believed live surgery to be a great way to educate(34), and 90% of orthopaedists considered the educational benefit to be good or very good(18). 75% of urological surgeons thought the audience desired a safe and slick demonstration(17) and, in keeping with this, 65.3% of ophthalmologists consider the demonstration of unedited surgical practice to be an advantage of live surgery(15). Participants at the seventh international meeting on reconstructive urology (IMORU) valued improvements in knowledge regarding surgical technique(35). Ackermann et al. found the benefits of live surgery on real patients to be ‘very high’ for surgical education and training, avoiding complications, learning innovative techniques, and improving surgical skills. Although ‘very high’ ratings were also received for the educational benefit of live surgery on body donors, the option of replacing real patients with donors at live surgery events was rated ‘very low’(7).

Five studies compared the educational value between LSB and ALSB or edited video formats(12,14,20,27,36). 93% of urologists viewing live surgery described the learning experience to be extremely or very helpful, in comparison to 80% for unedited videos and 69% for edited videos(12). Similarly, Schuettfort et al. reported higher ratings for the educational value of live surgery compared to semi-live surgery(36). However, Legemate et al. found live and semi-live surgery to be equally valuable in both learning how to manage complications and learning new tips and tricks(20). Likewise, Finch et al. found the educational value to be similar and no perceived difference in learning about the management of surgical complications, but a greater likelihood of learning tips and tricks from the surgeon during ALSB(14). In keeping with this, post-conference surveys from Phan et al. found that 54.5% of respondents regarded ALSB videos to have more educational value than LSB and 78.2% of respondents perceived that ALSB teaching was more time-efficient than LSB(27).

Four studies assessed the benefit of live streaming surgical procedures for large group teaching, as an alternative to traditional teaching methods(13,21,24,39). 61.5% of students reported a subjective gain in knowledge(39), 92% described the learning experience as very or extremely helpful, with a particular benefit for clinical and anatomical knowledge(24), and students left with fewer unanswered questions(21). Interaction between faculty and participants was viewed positively(13), for example: 76% found the surgeon and in-room facilitator very or extremely helpful(24) and, in direct comparison with an operating room cohort, students being taught by teleconference asked and were asked more than 4 times as many questions(21). Livestreams received high ratings(13), 88% would attend similar teaching again(24) 83% felt live-surgery should be an integral part of teaching and 89.2% asked for an increased offer of live-stream surgery(39).

## The future of live surgery

Survey results on the perceived value of live versus semi-live surgery are discordant. Schuettfort et al. found 90% of participants favoured live surgery over semi-live surgery(36) and Salami et al. reported 95.2% of respondents would participate in another LSB, with only 35.7% preferring unedited or edited videos over LSBs(34).

However, 64.2% of ophthalmologists think live surgery should be discontinued(15), with only 30.1% of urologists agreeing that LSBs should continue indefinitely in their present form and 55.4% in favour of replacement of live surgery with simulation technology once it improves(11). 60.3% of urologists believed ALSB to be superior to LSB in a post-conference survey(27) and 68.3% of ophthalmologists thought live surgery was no more beneficial than a video(15). Legemate et al. highlight that a balance must be found; 82% of respondents would like to see more semi-live surgery, but 33% would attend fewer demonstrations if semi-live surgery were to replace live surgery(20).

**Supplementary Figures and Tables**

**Table S1: Inclusion and exclusion criteria using PICOS framework.**

| Domain | Inclusion Criteria | Exclusion Criteria |
| --- | --- | --- |
| Population | Surgical trainees or qualified surgeons of any specialty.  Medical students.  Patients undergoing live surgical procedures. | Other healthcare workers. |
| Intervention | Any surgical intervention performed live and simultaneously broadcast, including those performed on living humans or cadavers. | Interventional or endoscopic procedures not performed by surgeons.  Non-surgical procedures.  Remote live surgery or proctoring.  Live lectures and/or virtual learning.  Veterinary procedures. |
| Comparison | N/A | N/A |
| Outcome | Clinical outcomes of surgery.  Benefit for the audience.  Opinions of surgeons, patients or viewers. | The quality of video/audio and/or broadcast related outcomes. |
| Study Type | Primary studies written in English with the full-text available.  Systematically performed reviews of clinical studies. | Studies published before January 1st 2005.  Studies not written in English, where no translation was available.  Case reports, abstracts, narrative reviews, guidelines, protocols, expert opinion, basic science studies, animal studies.  Overlapping studies on the same patient population (most recent included). |

**Table S2: Detailed search strategy. Databases searched: Ovid EMBASE, MEDLINE, and PubMed. Filters: ti = title, ab = abstract, af = all fields.**

| Live variations | Surgery variations | Outcome variations |  |
| --- | --- | --- | --- |
| 1) live | 8) surg* | 14) outcome | 20) 7 AND 13 AND 19 |
| 2) livestreaming | 9) resect* | 15) complication |  |
| 3) live-streaming | 10) incision | 16) consequence |  |
| 4) livestream | 11) operation | 17) effect |  |
| 5) live-stream | 12) operating theatre | 18) end result |  |
| 6) demonstration |  |  |  |
| 7) (1 OR 2 OR 3 OR 4 OR 5 OR 6) ti,ab. | 13) (8 OR 9 OR 10 OR 11 OR 12) af. | 19) (14 OR 15 OR 16 OR 17 OR 18) af. |  |

**Table S3: Included primary studies. Categorisation of included papers with overlap between categories. + denotes that the domain was included in the study.**

| **Study** | **Impact on patients** | **Impact on the audience** | **Impact on surgeons** | **Alternatives to live surgery** |
| --- | --- | --- | --- | --- |
| Ackermann et al.(7) |  | + |  | + |
| Altmann et al.(8) | + |  |  |  |
| Andolfi and Gundeti(9) | + |  |  |  |
| Dingemann et al.(10) | + | + | + |  |
| Duty et al.(11) | + |  | + | + |
| Elsamra et al.(12) | + | + | + | + |
| Feenstra et al.(13) |  | + |  |  |
| Finch et al.(14) | + | + | + |  |
| Hollick and Allan(15) | + | + | + |  |
| Itam et al.(16) | + |  |  |  |
| Khan et al.(17) | + | + | + |  |
| Lafosse et al.(18) | + | + |  |  |
| Legemate et al. (2017)(19) | + |  |  |  |
| Legemate et al. (2018)(20) | + | + | + | + |
| McIntyre et al.(21) | + | + |  |  |
| Misrai et al.(22) | + |  |  |  |
| Mullins et al.(23) | + |  |  |  |
| O’Brien et al.(24) |  | + |  |  |
| Ogaya-Pinies et al.(25) | + |  |  |  |
| Ozman et al.(26) | + |  |  |  |
| Phan et al.(27) | + | + |  |  |
| Puzo et al.(28) | + |  |  |  |
| Ramírez-Backhaus et al.(29) | + |  |  |  |
| Ratti et al.(30) | + |  |  |  |
| Rocco et al.(31) | + |  |  |  |
| Roman et al.(32) | + |  |  |  |
| Ruiz de Gordejuela et al.(33) | + |  |  |  |
| Salami et al.(34) | + | + | + | + |
| Schuettfort et al. (2019)(35) | + | + |  |  |
| Schuettfort et al. (2021)(36) |  | + |  | + |
| Seeburger et al.(37) | + |  |  |  |
| Unal et al.(38) | + |  |  |  |
| Van Bonn et al.(39) |  | + |  |  |

**Table S4: The impact of live surgery by domain.**

| **Impact on patients** | **Impact on the audience** | **Impact on surgeons** |
| --- | --- | --- |
| (Potential) risk of patient harm  Ethical issues  Adequate consent  Patient confidentiality  Treatment delays  Issues around continuity of care and management of potential complications  Patient benefit: expert surgeons, innovative methods, expenses covered | Educational benefit: real-time demonstration of novel techniques, management of intraoperative complications, tips and tricks, and improving surgical skills.  Increased student engagement in group teaching: improvements in knowledge gained and fewer unanswered questions. | Autonomy over operation  Pressure and anxiety  Distraction by audience  Simultaneous narration and interaction while performing surgery  Unfamiliar team and equipment  Communication problems  Jet lag |

**Table S5: Outcomes of retrospective studies on risk to live surgery patients. Complication rates, length of hospital stay, and operative time are compared to either the available literature or a control group used in the study.**

| **Study** | **Specialty** | **Number of live cases** | **Comparator** | **Complication rate** | **Length of Hospital stay** | **Operative time** | **Other findings** |
| --- | --- | --- | --- | --- | --- | --- | --- |
| Altmann et al. | Urogynaecology | 69 | Literature | Comparable to literature (no difference) | Within normal range. Mean stay was 8 days. |  |  |
| Andolfi and Gundeti | Paediatric Urology | 22 | Literature | Comparable to literature (no difference) |  |  |  |
| Itam et al. | Urology and Urogynaecology | 62 | Literature | Comparable to literature (no difference) |  |  |  |
| Rocco et al. | Urology | 224 | Literature | Acceptable (no difference) |  |  |  |
| Schuettfort et al. (2019) | Urology | 57 | Literature | Acceptable (no difference) |  |  | Revision surgery necessary in 3.5% of cases |
| Seeburger et al. | Cardiac | 250 | Literature | Comparable to literature (no difference) |  |  | Estimated survival of 90.5% at 10 years using Kaplan Meier analysis |
| Lafosse et al. | Orthopaedics (Shoulder) | 179 | Literature | Lower than literature |  |  |  |
| Ruiz de Gordejuela et al. | Bariatric | 107 | Literature | Higher than literature |  |  | 4.7% of patients required revision surgery. |
| Legemate et al. (2017) | Urology | 151 | Control group (n=697) | No difference | No difference | Operative time was longer for ureterorenoscopic live surgery group but there was no difference for the percutaneous group. |  |
| Misrai et al. | UrologyUrology | 37 | Control group (n=89) | No difference | No difference | No difference |  |
| Mullins et al. | Urology | 39 | Control group (n=847) | No difference | No difference | No difference | No difference in positive surgical margin rate for robotic partial nephrectomy |
| Ogaya-Pinies et al. | Urology | 36 | Control group (n=108) | No difference |  | Median console time was shorter for the live surgery group (73 min versus 78 min) |  |
| Ozman et al. | Urology | 24 | Control group (n=24) | No difference |  |  |  |
| Ramírez-Backhaus et al. | Urology | 23 | Control group (n=46) | No difference | No difference | No difference | No difference in intraoperative blood loss.  Higher rate of positive margins in live cases. |
| Puzo et al. | Ophthalmology | 54 | Control group (same individuals, opposite eye, n=54) | No difference |  |  | Greater loss of endothelial cell density in live surgery using toric intraocular lenses compared to non live surgery using toric intraocular lenses. |
| Ratti et al. | Hepatic | 60 | Control group (n=180) | No difference | No difference | Live surgeries required longer operating time (280 min versus 210 min) | No differences in intraoperative blood loss, conversion to open rate and achievement of R0 surgical margins. |
| Roman et al. | Colorectal | 33 | Control group (n=780) | No difference |  | No difference |  |
| Unal et al. | Colorectal | 39 | Control group (n=39) | Higher | Live surgery patients stayed longer in hospital (median of 6 days versus 5 days) | Operative time was longer (median 200 minutes versus 165 minutes) | Higher intraoperative estimated blood loss in live surgery group |

###

**Table S6: Effect of live surgical demonstrations on the experience of the performing surgeon.**

| **Paper** | **Study group (number of participants)** | **Effect on individual performance** | **Appropriateness of procedure** | **Challenges inside the operating room** | **Challenges outside the operating room** |
| --- | --- | --- | --- | --- | --- |
| Duty et al. | Members of the American Association of Genitourinary Surgeons (AAGUS) (n= 90) | **Anxiety levels (home/away institution):**  Moderate: 28.2%/ 29.8% High: 9.9%/ 25.0% Very high: 8.5%/ 17.9% | 43.9% often, 13.4% always concerned about the appropriateness.  20% had demonstrated a technique even though they would have chosen a different treatment. | 48.2% reported missing surgical equipment.  17.1% felt medical translation was a barrier.  16.9% reported inadequate surgical assistants. |  |
| Dingemann et al. | Members of the International Pediatric Endosurgery Group (n=61) | 83% of surgeons indicated elevated stress levels during live surgery. |  |  |  |
| Elsamra et al. | Attendees of American Urological Association national meeting 2012 and the second International Meeting of Challenges in Endourology and Functional Urology (n=253) |  |  |  | 26% felt that discussion can lead to surgeon distraction, increasing patient morbidity. |
| Hollick and Allan | UK consultant ophthalmologists (n=536) | 92.2% thought live surgery placed greater stress on the surgeon |  | 90.9% thought unfamiliar theatres, equipment, and staff to be a disadvantage of live surgery |  |
| Khan et al. | Attendees of the European Association of Urology Robotic Urology Society congress (n= 106) | **Anxiety levels versus no broadcast (home/away institution):**  No change: 18.3%/ 14%  Somewhat anxious: 62.4%/ 63.4%  Significant anxiety: 6.5%/ 19.4%  **Performance versus no broadcast (home/away institution)**  Better: 11.8%/ 4.3%  Same: 69.9%/ 67.4%  Worse: 18.3%/ 27.2%  Jet lag was found to be an important factor for 7.3% and contributing factor for 25% of surgeons. |  | 82.7% felt the surgical team from ‘foreign’ institutions was adequate.  22.8% brought their own surgical team in all or most cases.  59.8% reported occasionally lacking equipment  50% reported language or communication barriers. |  |
| Salami et al. | Faculty who had performed or moderated a live case demonstration at any World Congress of Endourology meeting from 2008 to 2012 (n=92) | **Anxiety levels (home/away institution):**  Moderate to very high: 34.6%/ 79.8% | 30% reported the selection of an appropriate case by the host organisation.  75.6% reported appropriate technical complexity of the procedure, 22% reported this as slightly too difficult. | 67.5% considered an unfamiliar team a distraction.  66.2% considered unfamiliar equipment a distraction. | 45.7% found moderator and audience discussions to be a distraction.  43.2% found narrating to the audience to be a distraction. |
| **Semi-live or As-live surgery** | | | | | |
| Finch et al. | Attendees of the 2013 Societe Internationale d’Urologie UK meeting (n=62) and the 2014 British Association of Urological Surgeons Endourology meeting (n=103) | Respondents perceived the surgeon to be less pressurised/anxious during an as-live broadcast compared to a live surgical broadcast. |  |  |  |
| Legemate et al. (2018) | Attendees of the 2017 Challenges in Endourology congress (n=279) | Faculty opinion of how often anxiety affected performance during a LSB (home/away institution)  Almost always: 20%/ 26%  Rarely: 61%/60%  Audience opinion of how often anxiety affected performance (LSB vs ALSB)  Almost always or often: 36%/ 9%  13% noted jet lag almost always or often negatively affected performance during live surgery. |  | 33% of faculty believed unfamiliarity with the surgical team or equipment often or almost always negatively affects performance during LSB. | 23% of respondents felt surgeons were often or almost always distracted by the audience during LSB.  Distraction by the audience during surgery is greater during LSB than ALSB. |

###

**References**

1. Tricco AC, Lillie E, Zarin W, O’Brien KK, Colquhoun H, Levac D, et al. PRISMA Extension for Scoping Reviews (PRISMA-ScR): Checklist and Explanation. Ann Intern Med. 2018 Oct 2;169(7):467–73.

2. Arksey H, O’Malley L. Scoping studies: towards a methodological framework. Int J Soc Res Methodol. 2005 Feb 1;8(1):19–32.

3. Methley AM, Campbell S, Chew-Graham C, McNally R, Cheraghi-Sohi S. PICO, PICOS and SPIDER: a comparison study of specificity and sensitivity in three search tools for qualitative systematic reviews. BMC Health Serv Res [Internet]. 2014 [cited 2023 Sep 16];14. Available from: https://www.ncbi.nlm.nih.gov/pmc/articles/PMC4310146/

4. Carbonara U., Crocerossa F., Novara G., Ditonno P., Pansadoro V., Breda A., et al. Risks and Benefits of Live Surgical Broadcast: A Systematic Review. Eur Urol Focus. 2022;8(3):870–81.

5. Awad M, Chowdhary M, Hermena S, Falaha SE, Slim N, Francis NK. Safety and effectiveness of live broadcast of surgical procedures: systematic review. Surg Endosc. 2022;36(8):5571–94.

6. Brunckhorst O., Challacombe B., Abboudi H., Khan M.S., Dasgupta P., Ahmed K. Systematic review of live surgical demonstrations and their effectiveness on training. Br J Surg. 2014;101(13):1637–43.

7. Ackermann J, Wedel T, Holthaus B, Bojahr B, Hackethal A, Brucker S, et al. Didactic Benefits of Surgery on Body Donors during Live Surgery Events in Minimally Invasive Surgery. J Clin Med. 2020 Sep;9(9):2912.

8. Altmann J., Chekerov R., Fotopoulou C., Muallem M.-Z., Du Bois A., Cliby W., et al. Ten years of live surgical broadcast at Charite-MAYO conferences (2010-2019): a systematic evaluation of the surgical outcome. Int J Gynecol Cancer. 2022;32(6):746–52.

9. Andolfi C, Gundeti MS. Live-case demonstrations in pediatric urology: Ethics, patient safety, and clinical outcomes from an 8-year institutional experience. Investig Clin Urol. 2020 Feb;61(Suppl 1):S51–6.

10. Dingemann J., Laje P., St. Peter S.D., Ure B.M. IPEG survey on live case demonstrations in pediatric surgery. J Laparoendosc Adv Surg Tech. 2012;22(7):705–9.

11. Duty B., Okhunov Z., Friedlander J., Okeke Z., Smith A. Live surgical demonstrations: An old, but increasingly controversial practice. Urology. 2012;79(5):e7-1185.

12. Elsamra SE, Fakhoury M, Motato H, Friedlander JI, Moreira DM, Hillelsohn J, et al. The surgical spectacle: a survey of urologists viewing live case demonstrations. BJU Int. 2014;113(4):674–8.

13. Feenstra TM, van Felius LP, Vriens MR, Stassen LPS, van Acker GJD, van Dijkum EJMN, et al. Does livestreaming surgical education have the future? Development and participant evaluation of a national COVID-19 livestreaming initiative. Surg Endosc. 2022;36(8):6214–22.

14. Finch W, Masood J, Buchholz N, Turney BW, Smith D, Wiseman O. Would You Want to Be the Patient? ‘Live Surgical Broadcast’ or ‘As-Live Unedited Surgical Broadcast’. J Endourol. 2015 Jul;29(7):821–9.

15. Hollick EJ, Allan BD. Live surgery: national survey of United Kingdom ophthalmologists. J Cataract Refract Surg. 2008 Jun;34(6):1029–32.

16. Itam S, Pakzad M, Hamid R, Ockrim J, Vashisht A, Cutner A, et al. Female Urology and Urogynecology: The Outcome of Patients Participating in Live Surgical Broadcasts. Urogynecology. 2020 Sep;26(9):554.

17. Khan SAA, Chang RTM, Ahmed K, Knoll T, van Velthoven R, Challacombe B, et al. Live surgical education: a perspective from the surgeons who perform it. BJU Int. 2014 Jul;114(1):151–8.

18. Lafosse L, Protais M, Moody MC, Colas M, Puah KL, Lafosse T. Live Surgery: A retrospective study on the outcomes and complications of 7 orthopedic live surgery events. Orthop Traumatol Surg Res. 2021 Dec 1;107(8):102871.

19. Legemate JD, Zanetti SP, Baard J, Kamphuis GM, Montanari E, Traxer O, et al. Outcome from 5-year live surgical demonstrations in urinary stone treatment: are outcomes compromised? World J Urol. 2017 Nov;35(11):1745–56.

20. Legemate JD, Zanetti SP, Freund JE, Baard J, de la Rosette JJMCH. Surgical teaching in urology: patient safety and educational value of ‘LIVE’ and ‘SEMI-LIVE’ surgical demonstrations. World J Urol. 2018 Oct;36(10):1673–9.

21. McIntyre TP, Monahan TS, Villegas L, Doyle J, Jones DB. Teleconferencing Surgery Enhances Effective Communication and Enriches Medical Education. Surg Laparosc Endosc Percutan Tech. 2008 Feb;18(1):45.

22. Misrai V., Guillot-Tantay C., Pasquie M., Bordier B., Guillotreau J., Gomez-Sancha F., et al. Comparison of Outcomes Obtained After Regular Surgery Versus Live Operative Surgical Cases: Single-centre Experience with Green Laser Enucleation of the Prostate. Eur Urol Focus. 2019;5(3):518–24.

23. Mullins JK, Borofsky MS, Allaf ME, Bhayani S, Kaouk JH, Rogers CG, et al. Live Robotic Surgery: Are Outcomes Compromised? Urology. 2012 Sep 1;80(3):602–7.

24. O’Brien J.W., Natarajan M., Aryal K. LIVE streaming of laparoscopic general surgery is a useful teaching tool: Results from a medical student survey. Surg Endosc. 2018;32(Supplement 2):S448.

25. Ogaya-Pinies G, Abdul-Muhsin H, Palayapalayam-Ganapathi H, Bonet X, Rogers T, Rocco B, et al. Safety of Live Robotic Surgery: Results from a Single Institution. Eur Urol Focus. 2019 Jul;5(4):693–7.

26. Ozman O, Citgez S, Basatac C, Akgul M, Yazıcı CM, Tanidir Y, et al. Safety of Live Retrograde Intrarenal Surgery; Results from a Boutique Course Series [Internet]. Preprints; 2020 Nov [cited 2023 Aug 14]. Available from: https://www.authorea.com/users/375408/articles/495944-safety-of-live-retrograde-intrarenal-surgery-results-from-a-boutique-course-series?commit=f26d8f2b9620d777b51f2b5de95e817e9bbff68e

27. Phan YC, Segaran S, Wiseman O, James P, Clayman R, Smith A, et al. Which Is Better? “Live” Surgical Broadcasts vs “As-Live” Surgical Broadcasts. J Endourol. 2016 Sep;30(9):1022–8.

28. Puzo P, D’Oria F, Imburgia A, Incandela C, Sborgia A, Marchegiani EB, et al. Live surgery outcomes in cataract surgery. Eur J Ophthalmol. 2022 Nov 1;32(6):3444–50.

29. Ramirez-Backhaus M., Bertolo R., Mamber A., Ferrer A.G., Mir M.C., Rubio-Briones J. Live Surgery for Laparoscopic Radical Prostatectomy-Does it Worsen the Outcomes? A Single-center Experience. Urology. 2019;123((Ramirez-Backhaus, Mamber, Ferrer, Mir, Rubio-Briones) Department of Urology, Fundacion IVO, Valencia, Spain):133–9.

30. Ratti F, Fiorentini G, Cipriani F, Paganelli M, Catena M, Aldrighetti L. Safety of minimally invasive liver resections during live surgery: a propensity score based assessment. HPB. 2019 Mar 1;21(3):328–34.

31. Rocco B, Grasso AAC, De Lorenzis E, Davis JW, Abbou C, Breda A, et al. Live surgery: highly educational or harmful? World J Urol. 2018 Feb 1;36(2):171–5.

32. Roman H, Prosszer M, Marabha J, Merlot B, Forestier D, Noailles M, et al. Live surgery of colorectal endometriosis broadcasted from a surgeon’s routine operating theater is not associated with higher complications rate. Acta Obstet Gynecol Scand. 2021;100(12):2176–85.

33. Ruiz de Gordejuela A.G., Ramos A.C., Neto M.G., Nora M., Torres Garcia A.J., Sanchez Pernaute A., et al. Live surgery courses: retrospective safety analysis after 11 editions. Surg Obes Relat Dis. 2018;14(3):319–24.

34. Salami SS, Elsamra SE, Motato H, Leavitt DA, Friedlander JI, Paoli MA, et al. Performing in the Surgical Amphitheater of Today: Perception of Urologists Conducting Live Case Demonstrations. J Endourol. 2014 Sep;28(9):1121–6.

35. Schuettfort V.M., Schoof J., Rosenbaum C.M., Ludwig T.A., Vetterlein M.W., Leyh-Bannurah S.-R., et al. Live surgery in reconstructive urology: evaluation of the surgical outcome and educational benefit of the international meeting on reconstructive urology (IMORU). World J Urol. 2019;37(11):2533–9.

36. Schuettfort VM, Ludwig TA, Marks P, Vetterlein MW, Maurer V, Fuehner C, et al. Learning benefits of live surgery and semi-live surgery in urology—informing the debate with results from the International Meeting of Reconstructive Urology (IMORU) VIII. World J Urol. 2021;39(7):2801–7.

37. Seeburger J., Diegeler A., Dossche K., Lange R., Mohr F.W., Schreiber C., et al. Live broadcasting in cardiac surgery does not increase the operative risk. Eur J Cardiothorac Surg. 2011;40(2):367–71.

38. Unal UK, Esen E, Yilmaz BS, Aytac E, Bilgin IA, Ozben V, et al. Live surgical demonstrations for minimally invasive colorectal training. Langenbecks Arch Surg. 2020 Feb 1;405(1):63–9.

39. van Bonn SM, Grajek JS, Schneider A, Oberhoffner T, Mlynski R, Weiss NM. Interactive live-stream surgery contributes to surgical education in the context of contact restrictions. Eur Arch Otorhinolaryngol. 2022 Jun 1;279(6):2865–71.
